# Expression of MMP-2 and TIMP-1 in Renal Tissue of Patients with Chronic Active Antibody-mediated Renal Graft Rejection

**DOI:** 10.1186/1746-1596-7-141

**Published:** 2012-10-12

**Authors:** Qiang Yan, Weiguo Sui, Baoyao Wang, Hequn Zou, Guimian Zou, Hao Luo

**Affiliations:** 1 Dialysis and Kidney Transplantation Center of PLA, 181 Hospital of PLA, Guilin, 541002, China; 2 Institute of Nephrology and Urology, Third Affiliated Hospital of Southern Medical University, Guangzhou, 510630, China; 3 First Clinical College of Southern Medical University, Guangzhou, 510515, China

**Keywords:** Matrix metalloproteinase-2, Tissue inhibitor of metalloproteinase-1, Chronic active antibody-mediate rejection, Interstitial fibrosis and tubular atrophy

## Abstract

**Objective:**

To investigate the expression of matrix metalloproteinase-2 (MMP-2) and tissue inhibitor of metallopropteinase-1 (TIMP-1) in the renal allografts of patients with chronic active antibody-mediated rejection (AMR), and to explore their role in the pathogenesis of AMR.

**Methods:**

Immunohistochemistry assay and computer-assisted image analysis were used to detect the expression of MMP-2 and TIMP-1 in the renal allografts with interstitial fibrosis and tubular atrophy (IF/TA) in 46 transplant recipients and 15 normal renal tissue specimens as the controls. The association of the expression level of either MMP-2 or TIMP-1 with the pathological grade of IF/TA in AMR was analyzed.

**Results:**

The expression of either MMP-2 or TIMP-1 was significantly increased in the renal allografts of the recipients as compared with the normal renal tissue (*P* < 0.05). MMP-2 expression tended to decrease, while TIMP-1 and serum creatinine increased along with the increase of pathological grade of IF/TA (*P* < 0.05). In IF/TA groups, the expression of TIMP-1 was positively correlated to serum creatinine level (r = 0.718, *P* < 0.05).

**Conclusions:**

It is suggested by the results that abnormal expressions of MMP-2 and TIMP-1 might play roles in the development of renal fibrosis in chronic AMR.

**Virtual Slides:**

The virtual slide(s) for this article can be found here: http://www.diagnosticpathology.diagnomx.eu/vs/1128474926172838

## Introduction

C4d was found to sedimentate in peritubular capillaries in renal allografts in 1993. Since then it was looked as a sensitive indicator to detect humoral rejection and was included in Banff 07 pathologic diagnostic criteria, therefore chronic active antibody-mediated rejection (AMR) was being paid more and more attention [1]. However the exact pathogenesis of that was not full elucidated. Renal fibrosis, including renal interstitial fibrosis and glomerular sclerosis, is the common pathological mechanism of various chronic kidney diseases including chronic renal allograft dysfunction (CRAD) resulted from AMR, and finally develops into end-stage renal disease (ESRD). Previous studies suggest that matrix metalloproteinase-2 (MMP-2) and tissue inhibitor of metalloproteinase-1 (TIMP-1) were important cytokines for extracellular matrix (ECM) synthesis and degradation, and the excess accumulation of ECM is the main pathological mechanism of fibrosis. So we studied the relationship of MMP-2 and TIMP-1 with renal interstitial fibrosis in renal allografts.

## Methods

### Patients

The renal biopsy samples were collected from kidney transplant patients with proteinuria and elevated serum creatine level from January 2006 to December 2010 in Guilin No.181 Hospital. 46 patients with clinical diagnosis of chronic allograft dysfunction were diagnosed as AMR. Among them 32 were males (age 45 ± 9 years) and 14 were females (age 42 ± 8 years). The duration after kidney transplantation were 1-9 years (mean time of 3.5 years), the mean level of serum creatine was 346.93 ± 178.37 μmol/, 24h urinary protein >0.5g/24h and urinary protein (1+ - 4+). The triple immunosuppressant treatment protocol was cyclosporine + mycophenolate mofetil + prednisone in 28 patients and tacrolimus + mycophenolate mofetil + prednisone in 17 patients and sirolimus + mycophenolate mofetil + prednisone in 1 patient. Before renal biopsy, color doppler ultrasound detection in renal allografts and serum drug concentration test were performed to exclude acute rejection, nephrotoxicity of immunosuppressant, obstruction/reflux of ureter, thrombosis or embolism in renal arteries or veins and other diseases. According to Banff 2007 [2] renal allograft pathological classification criteria, patients with positive C4d deposit in renal allograft were diagnosed as AMR. The donor and recipient were matched in ABO blood groups and two or more HLA antigens were matched. The result of lymphocytotoxicity test was less than 10% and the result of panel reaction antibody (PRA) was negative. The renal samples of 15 cases of control were collected from routine donor kidney biopsy before transplantation and there was no pathological manifestation. Informed consents were obtained from all patients that participated in the study. Informed consents were obtained from all patients that participated to the study. This study was performed under the supervision of Institutional Review Board of Southern Medical University, and abided the Helsinki Declaration on ethical principles for medical research involving human subjects.

### Pathological classification

According to Banff 09 [2] renal allograft pathological diagnostic classification criteria, patients with C4d positive (linear deposit of C4d in 50% peritubular capillary) were diagnosed as AMR. All of the recipients were divided into three groups (IF/TA-I, IF/TA-II and IF/TA-III) according to the Banff 09 pathological diagnostic classification standard based on the degree of interstitial fibrosis of allograft tissue: IF/TA-I group including 16 patients with mild interstitial fibrosis and renal tubular atrophy (less than 25 percent renal cortex was involved); IF/TA-II group including 14 patients with moderate interstitial fibrosis and renal tubular atrophy (26 percent to 50 percent renal cortex was involved); IF/TA-III group including16 patients with severe interstitial fibrosis and renal tubular atrophy (more than 50 percent renal cortex was involved).

### Immunohistochemisty examination of MMP-2 and TIMP-1

Immunohistochemisty assay with EnVision was used to detect the expression of MMP-2, TIMP-1 and C4d deposit in the renal allograft tissue. Sections of 3-μm-thick tissue were hydrated through graded ethanol, and endogenous peroxidase was blocked with 3% peroxide hydrogen. For MMP-2 test the section was treated with microwave for 15 minute, however for TIMP-1 test the section was treated with 10mmol/L of pH6.0 citrate buffer solution. The section was incubated respectively with each of the primary antibodies (mouse anti-human MMP-2 monoclonal antibody, 1:200, Maixin Biological Technology Development Co. Fujian, No. MAB-O244; rabbit anti-human TIMP-1 polyclonal antibody, working solution, Maixin Biological Technology Development Co. Fujian, No. RAB-0282; rabbit anti-human C4d polyclonal antibody, working solution, Santa Cruz, No. RAB-02415) overnight at 4°C. After washing with PBS 3 × 3 min, then incubated with second antibody (rabbit anti-mouse monoclonal antibody, Maixin Biological Technology Development Co. Fujian) for 30 min at 37°C, and then washed with PBS 3 × 3min. Then the sections were treated with DAB coloration and re-stained with hematoxylin.

### Image semi-quantitative analysis

Images were acquired by means of Leica DMR-X microscope coupled to a Leica DC500 digital camera (Leica, Wetzlar, Germany) and the image analysis system Quantimet Q550 (Leica Imaging Systems). Ten randomly selected discontinuous fields (400x) per kidney were evaluated, including tubulointerstitial in renal cortex, medulla and the conjunction region, (but not including glomulular and vessels). More than 60 tubules in each biopsy section were obeserved. The positive area was yellow staining and Image-Pro Plus software was used to quantify the integrated optical density. The ratio of positive area to total tubulointerstitial area (not including the area of tubular lumens) presented the relative amount of the substance expression in tubuloinstitium.

### Statistical analysis

Results were expressed as means ± SD. Statistical analysis was performed with SPSS 13.0 (SPSS, Chicago, IL). Measurement data was analyzed with one way ANOVA, and the relationship was analyzed with Spearman rank correlation. Statistic significance was set at *P* < 0.05 level.

## Results

### Expression of MMP-2 and TIMP-1

**Figure 1:**
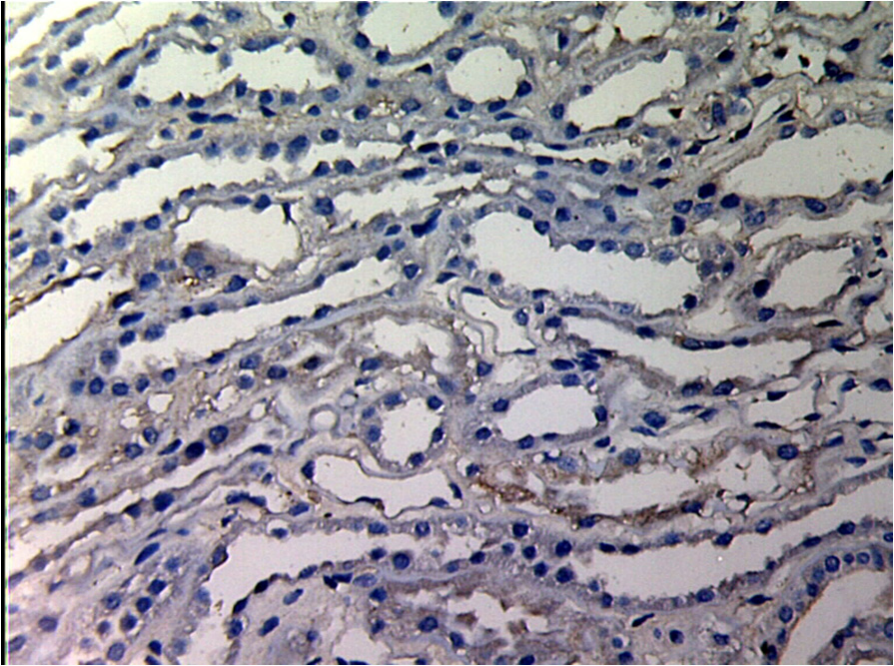
**MMP-2 expression in renal tissue with different degree of inflammatory infiltration(EnVision assay; original magnification × 200).** Control group.

**Figure 2:**
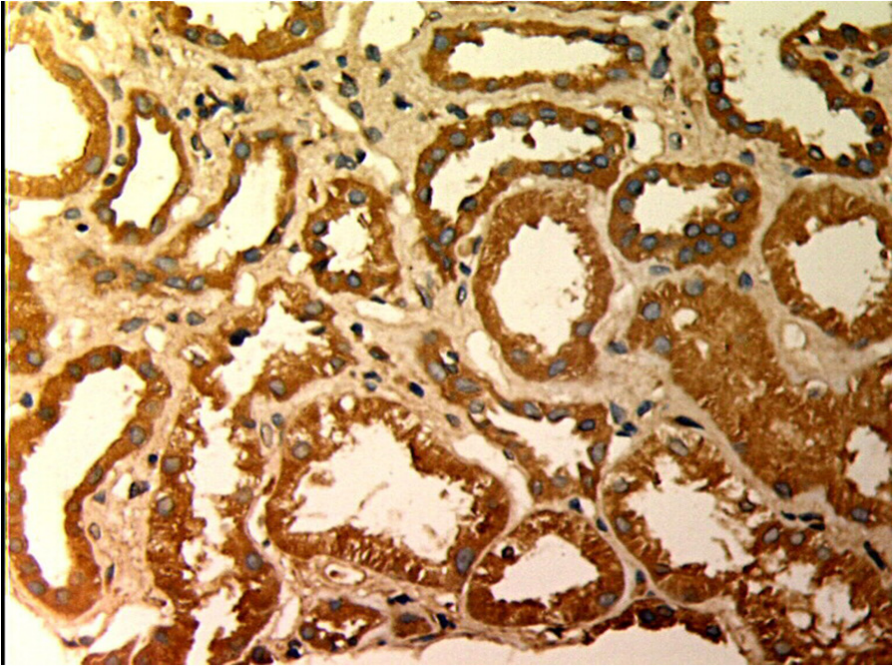
**MMP-2 expression in renal tissue with different degree of inflammatory infiltration(EnVision assay; original magnification × 200).** IF/TA-I Group.

**Figure 3:**
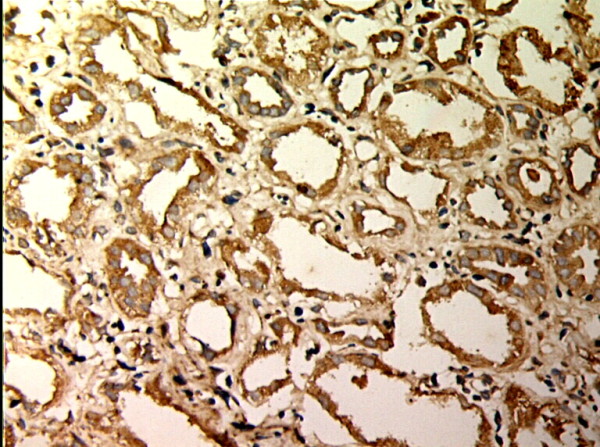
**MMP-2 expression in renal tissue with different degree of inflammatory infiltration(EnVision assay; original magnification × 200).** IF/TA-II Group.

**Figure 4:**
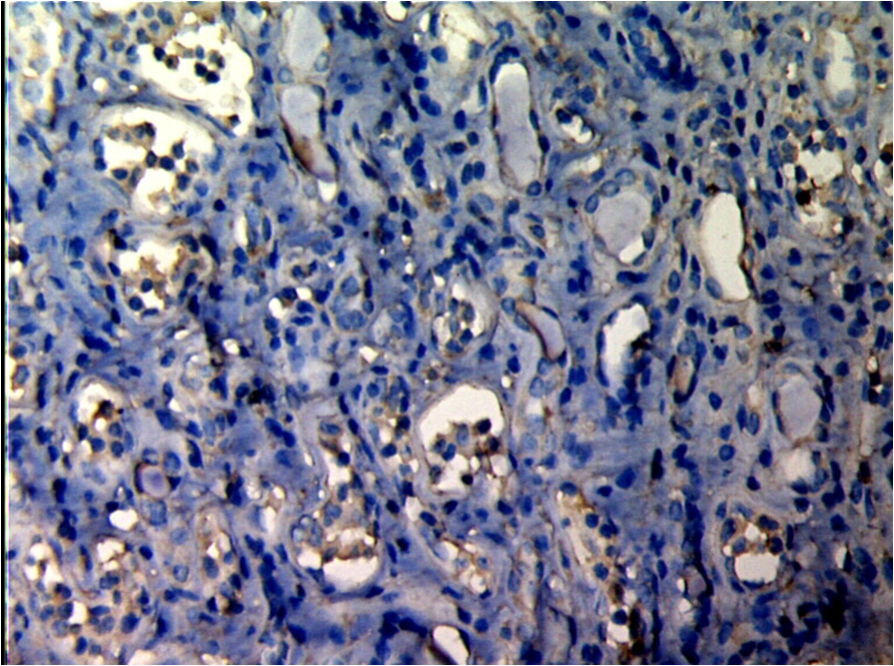
**MMP-2 expression in renal tissue with different degree of inflammatory infiltration(EnVision assay; original magnification × 200).** IF/TA-III Group.

**Figure 5:**
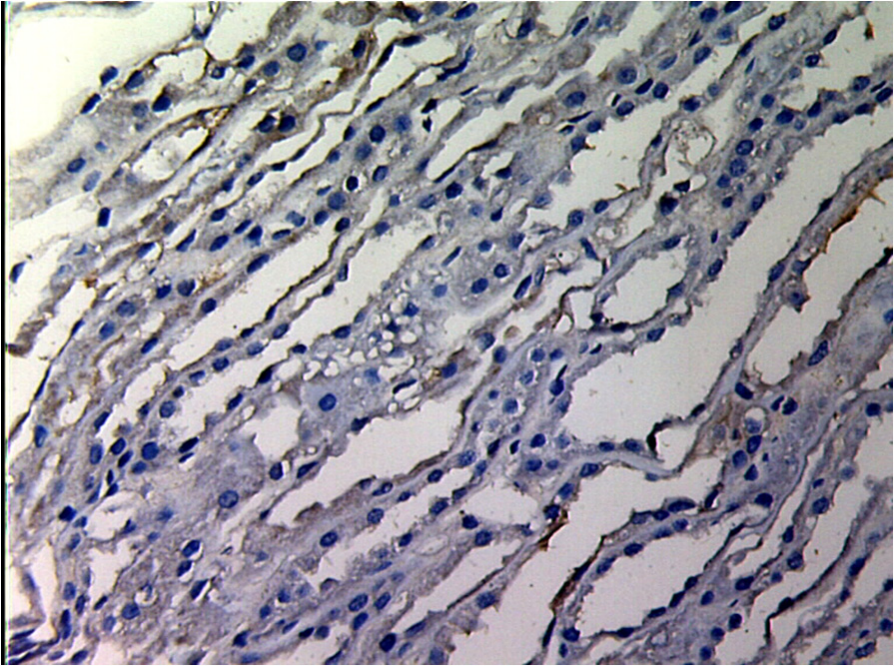
**TIMP-1 expressions in renal tissue with different degree of interstitial fibrosis/tubular atrophy.** (EnVision assay; original magnification × 200). Control Group

**Figure 6:**
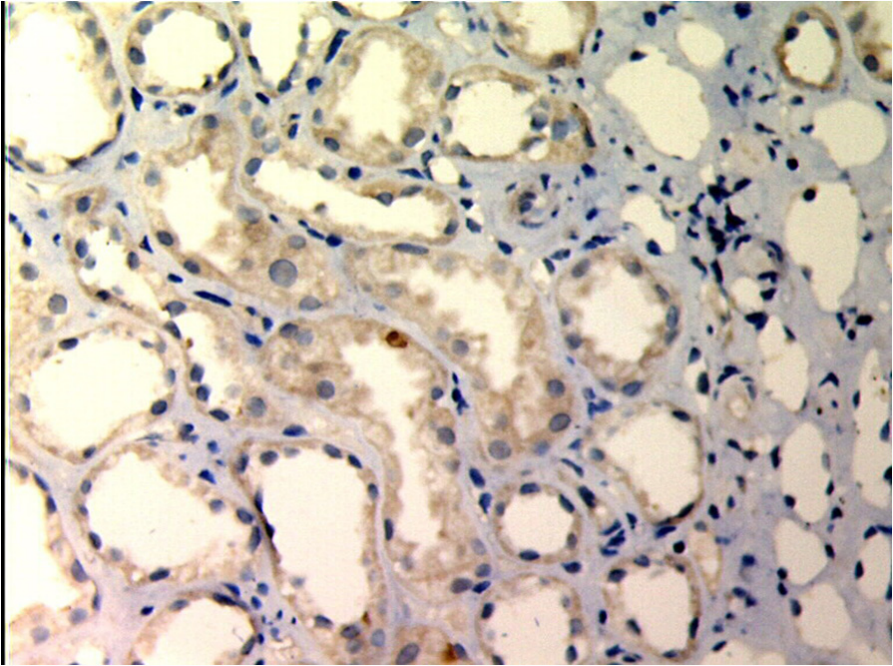
**TIMP-1 expressions in renal tissue with different degree of interstitial fibrosis/tubular atrophy.** (EnVision assay; original magnification × 200). IF/TA-I Group.

**Figure 7:**
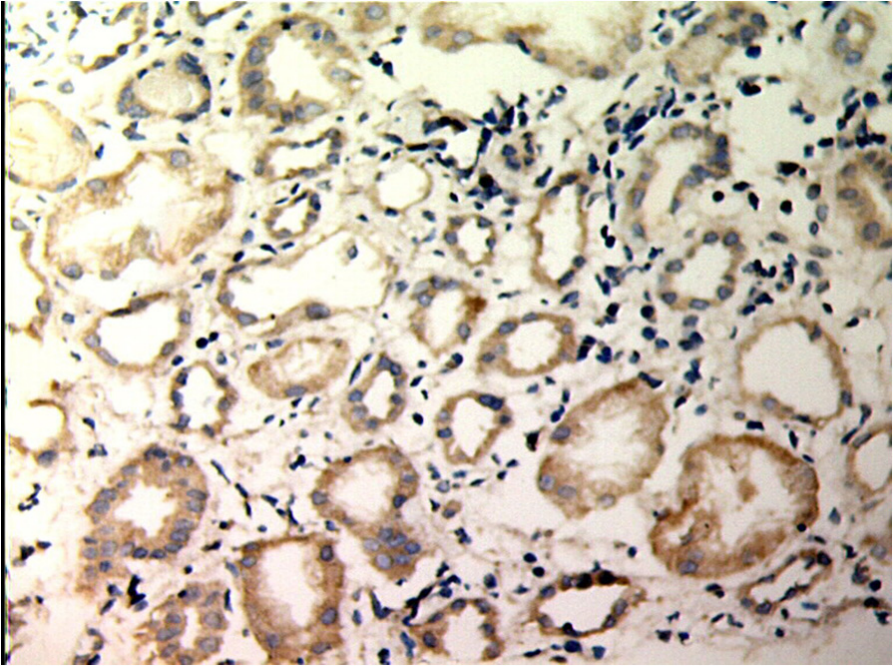
**TIMP-1 expressions in renal tissue with different degree of interstitial fibrosis/tubular atrophy.** (EnVision assay; original magnification × 200). IF/TA-II Group.

**Figure 8:**
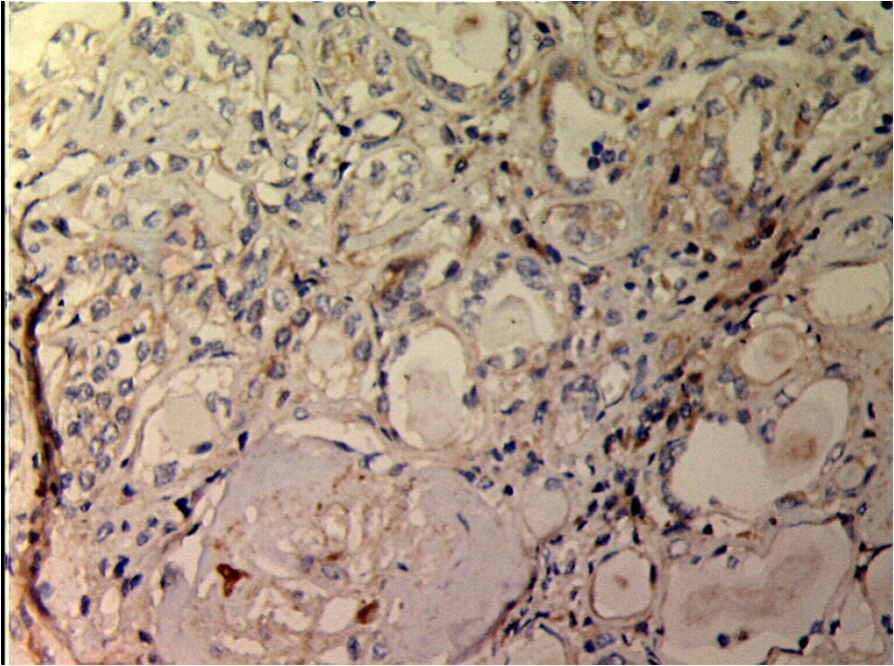
**TIMP-1 expressions in renal tissue with different degree of interstitial fibrosis/tubular atrophy.** (EnVision assay; original magnification × 200). IF/TA-III Group.

MMP-2 and TIMP-1 were mainly expressed in renal tubular epithelial cells, renal tubular basement membrane and cytoplasm of interstitial cells, looking like pale yellow, pale brown and russet particals as positive. There was no or rare expression of MMP-2 and TIMP-1 in normal kidney tissue, but there was significant expression of MMP-2 and TIMP-1 in AMR groups, mainly in tubular epithelial cells and cytoplasm of interstitial cells. MMP-2 was expressed to the most in IF-TA group, but decreased with the aggravation of mesenchyme disorder. However the expression of TIMP-1 increased with the aggravation of mesenchyme disease (Figures 1, 2, 3, 4, 5, 6, 7, 8). Expression of MMP-2 and TIMP-1 in tubular interstitial cell increased significantly in AMR groups compared to normal group, there was significant difference (*P* < 0.001), and expression of MMP-2 was decreaing with the increase of IF/TA pathological classification (*P* < 0.05), expression of TIMP-1 was increaing with the increase of IF/TA pathological classification (*P* < 0.05, Table 1).

**Table 1:** Expression of MMP-2 and TIMP-1 in the renal tissue and serum creatinine in groups with different degrees of interstitial fibrosis and tubular atrophy and normal controls (Mean ± SD)

**Groups**	**N**	**MMP-2 (%)**	**TIMP-1 (%)**	**Scrum (umol/L)**
Normal controls	15	8.10 ± 4.41	9.02 ± 5.64	87.32 ± 2.38
IF/TA-I	16	39.52 ± 8.91^※^	22.21 ± 12.62^※^	175.81 ± 41.53^※^
IF/TA-II	14	23.85 ± 5.25^※▲^	36.38 ± 13.12^※▲^	327.07 ± 102.14^※▲^
IF/TA-III	16	15.55 ± 4.16^※▲◆^	51.58 ± 12.75^※▲◆^	535.42 ± 123.20^※▲◆^

※:*P*<0.05, vs Control Group; ▲:*P*<0.05, vs IF/TA-IGroup; ◆:*P*<0.05. vs IF/TA-IIGroup.

### Expression of C4d

**Figure 9:**
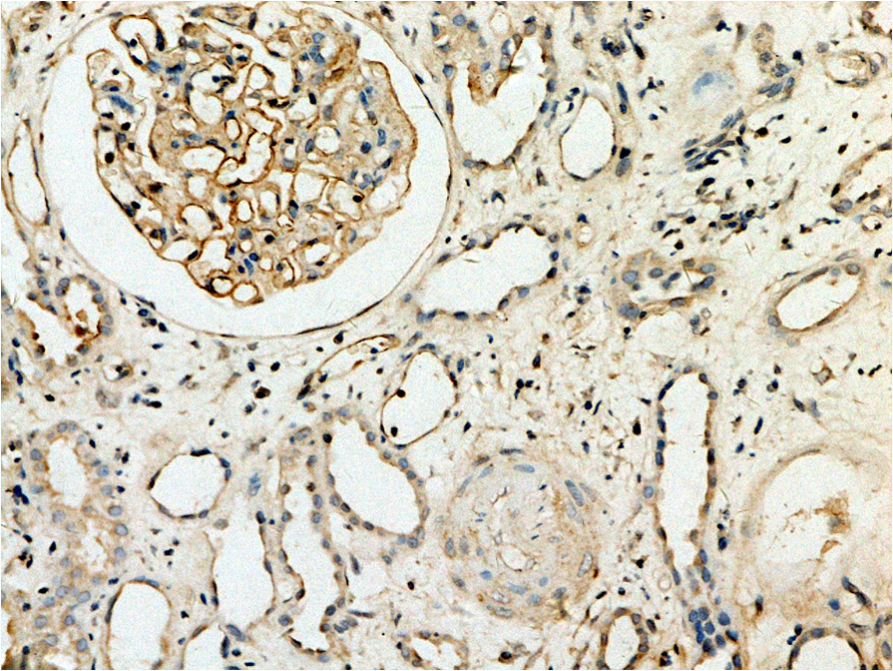
**Expression of C4d and renal histology.** C4d diffuse staining in glomerular and peritubular capillaries(EnVision assay; original magnification × 200).

**Figure 10:**
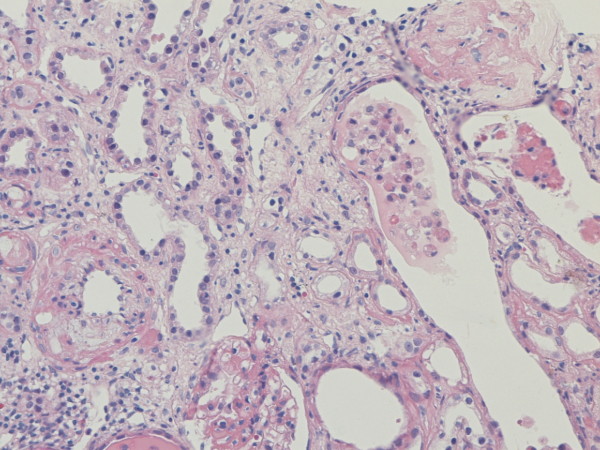
**Expression of C4d and renal histology.** Interstitial fibrosis/tubular atrophy (HE staining,original magnification × 200).

**Figure 11:**
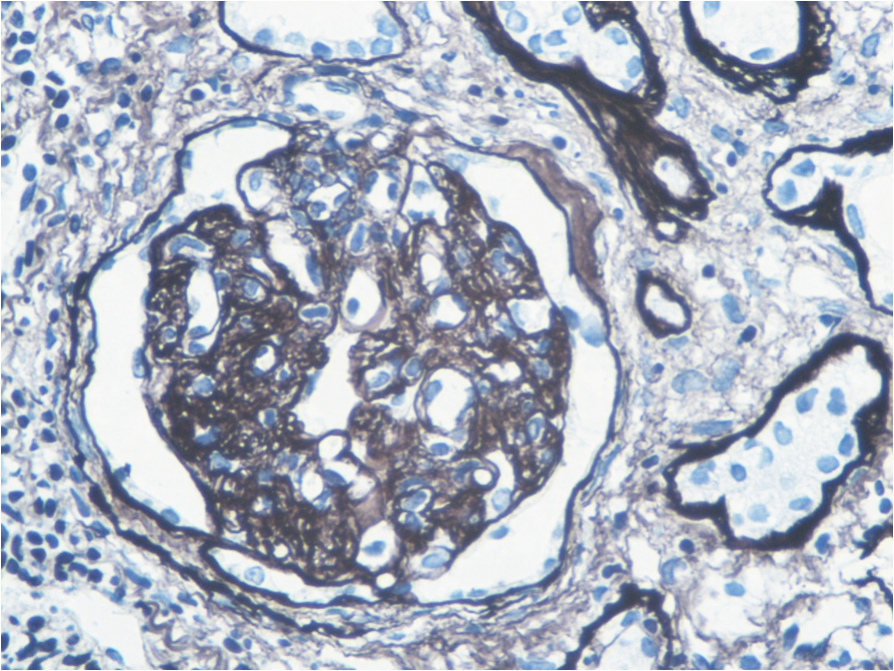
**Expression of C4d and renal histology.** Glomerular double contours (original magnification × 400).

Rare C4d deposits were found in endothelial cell and/or basal membrane of peri-tubular capillaries in normal kidney tissue, however diffuse and brightening positive linear deposits of C4d of 50% peri-tubular capillaries in AMR. The deposit of C4d in 50% peri-tubular capillaries endothelial cells and basal membrane was significant morphological dis- orders of complement activation in AMR (Figures 9, 10, 11).

### The correlation between Scr and renal fibrosis

Scr was increasing with the aggravation of renal fibrosis in patients (Table 1). It was shown by the statistic analysis with Spearman rank correlation coefficience that there was negative correlation between Scr and the expression level of MMP-2 in renal allograft tissue (r = -0.077, *P* = 0.556 >0.05), however there were positive correlation between Scr in serum and expression level of TIMP-1 in renal allograft tissue (r = 0.718, *P* = 0.000<0.05).

## Discussion

Renal transplantation is the best therapy for end stage renal failure. It improves life quality and survival time of recipients compared to other therapies such as hemodialysis. In the past decades,with the increasing use of new kinds of immuno-suppressants and improvment of HLA matching, the incidence of early acute rejection in renal transplantation decreased significantly, while long-term renal graft survival remain to improve. AMR is a main immune cause of long-term renal graft survival. The histopathological changes of renal allograft morphologically appear as renal interstitial fibrosis, tubular atrophy and thickening of glomerular basement membrane; and/or diffuse mononuclear cell infiltration and fibrosis in interstitium; partial tubular atrophy, lumen expansion, arrange irregularly, thickening of basement membrane, partial medullary ductal ectasia of kidney allograft, common protein casts; focal and segmental glomerulosclerosis, even if thoroughly hyaline arteriolosclosis, mesangial region broading, chickening of basement membrane, visible tram line shadow (Figure 2C), arterial wall intima proliferation, narrowing of artery, the intimal concentric and fibrous proliferation, stricture or blocking of lumen of interlobular artery or its branches (Figure 2B). The clinical manifestations were slowly creeping upward of serum creatinine, proteinuria and hypertention, eventually developing into ESRD, due to mesangial matrix sediment, thickening of glomerular basement membrane and renal interstitial fibrosis or tubular atrophy. The pathological chages involved into the disorder of ECM synthesis and break down. The synthesis of ECM was influenced by TGF and other cytokines, and the breakdown of ECM was influenced by MMPs and TIMPs.

MMPs, also called as collagenase IV or collagenase A, belong to one group of Zn dependent proteinases leading specific degradation of ECM, and one of their primary functions is to break down collagen IV. Collagen IV is the main constituent in glomerular basement membrane. MMP-2 with relative molecule weight 72,000, a kind of collagenase A, may degrade collagen IV [3]. The disorder of MMP-2 expression might induce changes in tubular basement membrane structure, thus resulting to renal tubular epithelial cell transdifferentiation with tubular atrophy, fibrosis and renal dysfunction [4]. MMP-2 participates in the degradation of ECM, however TIMP-1 regulates its activity. MMP-2 and TIMP-1 play important roles in regulating the synthesis and degradation of ECM.

The results of our present study suggested that MMP-2 and TIMP-1 were weakly expressed in normal kidney tissue, but their expression was upregulated in AMR. MMP-2 was mainly expressed in IF/TA-I group, and its expression was decreasing along with the aggravation of pathological classification, however the expression of TIMP-1 was increasing along with the aggravation of the pathological classification of IF/TA, simultaneously renal function was gradually aggravated and the level of Scr was increasing along with the pathological classification of IF/TA. The imbalance between MMP-2 and TIMP-1 might participate in the development of renal allograft fibrosis, so it is necessary to explore the mechanism how the imbalance between MMP-2 and TIMP-1 participates in renal interstitial fibrosis and tubular atrophy of AMR.

It was reported in literature that the expression of MMP-2 was decreased by 25mmol/L glucose, but the expression of TIMP-2, the special inhibitor of TIMP-1, was upregulated, which promoted ECM sidiment in the cultured human glomerular epithelial cells [5]. Berthier CC et al [6] found that the expression of MMP-2 and its inhibitor TIMP-2 and mRNA of TGF increased significantly in chronic rejection animal model of Fisher and Lewis mouse. Experiment on unilateral ureteral obstruction model based on TIMP-1 transgenic mice found that the expression of TIMP-1 was upregulated, gelatinase activity was downregulated, ICAM-1 and TGF, collagenIand III were upregulated, renal interstitial fibrosis and macrophage infiltration aggravated, which suggest that TIMP-1 over expression might paiticipate in renal interstitial fibrosis and involve in the course of inflammatory reaction [7]. Wagrowska- Danilewicz et al [8] found that the expression of both MMP-2 and TIMP-1 were upregulated in reanal allografts of 17 chronic allograft nephropathy patients compared to renal tissue of 11 normal controls, which suggest the remodeling of impaired renal structure have correlation with the regulation imbalance of MMP-2 and TIMP-1. The expression of TIMP-1 was downregulated, while the mRNA and protein expression and activities of MMP-2 and MMP-9 were enhanced after treatment with benazapril and all-trans retinoic acid (ATRA) in Wistar rat model of glomerular sclerosis compared to normal group [9]. The results of above studies suggest the ATRA exerts protection role by downregulate the expression of TIMP-1 and upregulate the mRNA expression and activity of MMP-2 and MMP-9, regulate the balance of the ratio of MMPs and TIMPs, and thus decrease ECM sediment. However it remains a question which needs further study that how to inhibit the activity of TGF, keep the balance of the activities of MMPs and TIMPs, retard ECM over sediment and improve renal allograft function.

If further researches may determine the pathogenesis of how the imbalance of MMP-2/TIMP-1 participates in AMR, then it might be the target of avoiding chronic renal allograft dysfunction to regulate the expression and activities of MMP-2 and TIMP-1.

## Competing interests

The authors declare that they have no competing interest.

## Authors’ contributions

HZ and QY design the study, BW and GZ performed research and wrote the first draft of the manuscript, WS, HL participated in the statistical analyses. All the authors read and approved the final manuscript.
